# Time-Course Transcriptome Analysis of Compatible and Incompatible Pollen-Stigma Interactions in *Brassica napus* L.

**DOI:** 10.3389/fpls.2017.00682

**Published:** 2017-05-03

**Authors:** Tong Zhang, Changbin Gao, Yao Yue, Zhiquan Liu, Chaozhi Ma, Guilong Zhou, Yong Yang, Zhiqiang Duan, Bing Li, Jing Wen, Bin Yi, Jinxiong Shen, Jinxing Tu, Tingdong Fu

**Affiliations:** ^1^National Key Laboratory of Crop Genetic Improvement, National Center of Rapeseed Improvement in Wuhan, Huazhong Agricultural UniversityWuhan, China; ^2^Department of Leafy Vegetable, Wuan Institute of Vegetable ScienceWuhan, China

**Keywords:** time-course, self-(in) compatibility, stigma, transcriptome, *Brassica napus*

## Abstract

*Brassica* species exhibit both compatible and incompatible pollen-stigma interactions, however, the underlying molecular mechanisms remain largely unknown. Here, RNA-seq technology was applied in a comprehensive time-course experiment (2, 5, 10, 20, and 30 min) to explore gene expression during compatible/incompatible pollen-stigma interactions in stigma. Moderate changes of gene expression were observed both in compatible pollination (PC) and incompatible pollination (PI) within 10 min, whereas drastic changes showed up by 30 min, especially in PI. Stage specific DEGs [Differentially Expressed Gene(s)] were identified, and signaling pathways such as stress response, defense response, cell wall modification and others were found to be over-represented. In addition, enriched genes in all samples were analyzed as well, 293 most highly expressed genes were identified and annotated. Gene Ontology and metabolic pathway analysis revealed 10 most highly expressed genes and 37 activated metabolic pathways. According to the data, downstream components were activated in signaling pathways of both compatible and incompatible responses, and incompatible response had more complicated signal transduction networks. This study provides more detailed molecular information at different time points after compatible and incompatible pollination, deepening our knowledge about pollen-stigma interactions.

## Introduction

The proper interactions between pollen and stigma play a vital role in successful pollination which is the key process in reproduction for angiosperms. The *Brassicaceae* plants have evolved complicated and elaborate mechanisms for successful fertilization to produce vigorous progenies. These mechanisms involve blocking the adherence and growth of inter-species pollen, rejecting “self” pollen (self-incompatibility, SI) and only allowing the fertilization of compatible pollen with different genetic background. The *Brassicaceae* plants have dry stigmas (with no exudate) whose epidermis is composed of large specialized papillae cells covered by a waxy cuticle and a superficial proteinaceous pellicle layer (Elleman et al., 1988, 1992). Once compatible pollen lands on the stigma, a series of signaling events are triggered. During this process, a pollen grain experiences several steps, including adhesion, foot formation, pollen hydration, germination and penetration through the stigmatic cell walls. Following these steps, pollen tube grows down through the transmitting tissue of the style, and ultimately reaches an ovule where fertilization takes place (reviewed in Chapman and Goring, 2010). However, when “self” pollen lands on the stigma, the SI reaction occurs rapidly, blocking the self-compatible reaction from pollen adhesion to pollen tube penetration (reviewed in De Nettancourt, 2001; Franklin-Tong, 2008).

Several stigma specific genes have been shown to participate in compatible and incompatible pollen-stigma interactions in *Brassicaceae*. A stigma specific *S*-locus related-1 (*SLR1*) gene is involved in pollen adhesion, and knocking down of *SLR1* reduces pollen adhesion in *B. napus* (Luu et al., 1997). Another stigma specific protein, SLG (*S*-locus glycoprotein), could bind PCP-A1, a small pollen coat protein (Doughty et al., 1998). By treating *B. oleracea* stigmas with antibodies of SLG or SLR1 also reduced pollen adhesion (Luu et al., 1999). Samuel et al. (2009) reported that a non-stigma specific protein, EXO70A1, is required in the stigma for the acceptance of compatible pollen in both *Brassica* and *Arabidopsis* and is negatively regulated during SI in *Brassica*. In *Brassicaceae*, the SI reaction involves the interaction of SRK (*S*-locus receptor kinase) expressed in stigma and its pollen-coat localized ligand SCR/SP11 (*S*-locus cysteine-rich protein or *S*-locus protein 11) which is allele-specific, leading to autophosphorylation of SRK and triggering several signaling cascades within the stigma epidermal cells (Kachroo et al., 2001; Takayama et al., 2001). The phosphorylated SRK, together with the plasma membrane-tethered MLPK (M-locus Protein Kinase), can phosphorylate ARC1 (Armadillo Repeat-Containing protein 1), a U-box E3 ubiquitin ligase (Murase et al., 2004; Kakita et al., 2007a,b; Samuel et al., 2009). ARC1 is proposed to function in the proteasome-mediated degradation pathway, and it can target stigma proteins required for the compatible reaction (for example Exo70A1) (Samuel et al., 2011).

Knowledge about incompatible and compatible pollen-stigma interactions has increased considerably in recent years. In *B. rapa*, time-lapse imaging of pollen behavior during self- and cross-pollinations illustrates that pollen hydration is regulated by a balanced process of hydration, dehydration and nutrient supply to pollen grains from stigmatic papilla cells (Hiroi et al., 2013). Compatible pollination induces actin polymerization and leads to vacuolar rearrangements toward the pollen attachment site. During incompatible pollination, actin reorganizes (likely depolymerization) and disrupts vacuole networks toward the site of pollen attachment (Iwano et al., 2007). Safavian and Goring (2013) found that secretory activity was rapidly induced in stigmatic papillae by compatible pollen, with vesicle or multi-vesicular bodies (MVBs) observed at the stigmatic papillar plasma membrane under the pollen grain. In incompatible pollination the secretory activity was inhibited in *Brassicaceae*. Microarray technology and a cDNA library were used to build a profile of candidate stigma genes that facilitate early pollination events in *Arabidopsis* (Swanson et al., 2005). Through proteomic analysis of stigmatic proteins following incompatible pollination in *B. napus*, 19 down-regulated unique candidate proteins were identified specially in SI (Samuel et al., 2011). Matsuda et al. (2014) applied laser microdissection (LM) and RNA sequencing (RNA-seq) to detect the cell type-specific transcriptome in *Brassicaceae* papillae cells and characterized gene expression 1 h after compatible and incompatible pollination. Although these studies contributed to our understanding of the molecular mechanisms related to pollen-stigma interactions, the consecutive changes of gene expression and dynamic molecular activities during the early stages (within 30 min) of pollination remained to be revealed. In addition, compared with the intensive study of signal transduction pathways in hormones and disease resistance in *Brassicaceae*, the knowledge of downstream components in self-incompatibility is still quite limited.

Self- incompatibility of *B. napus* is regulated by the interaction between *BnSP11* and *BnSRK*, together with the activated downstream components following the interaction. *BnSRK* could recognize *BnSP11* specifically and get autophosphorylated, then phosphorylated SRK would phosphorylate ARC1 which could cause ubiquitination of its target proteins (e.g., Exo70A1). An insertion of a DNA fragment with a length of 3606 bp in the promoter region of *BnSP11-1* was responsible for the self-compatibility (SC) of *B. napus* line “Westar” (Okamoto et al., 2007; Tochigi et al., 2011). By complementing the function of *BnSP11-1* in “Westar,” we obtained the transgenic line “W-3” which showed strong SI (Gao et al., 2016). When pollen of “Westar” lands on its own stigma, compatible interaction occurs and normal pods are set; when pollen of “W-3” lands on the stigma of “Westar,” self-incompatible reaction occurs and pods seldom set seeds. These are ideal materials for us to shed further light on the complex responses of compatible/incompatible pollen-stigma interactions using next-generation RNA-seq coupled with a comprehensive time-course experiment.

## Materials and methods

### Plant material and growth conditions

The wild type self-compatible *B. napus* line “Westar” and transgenic self-incompatible line “W-3” (Gao et al., 2016) were grown in the greenhouse with 16/8 h day/night photoperiod and temperatures of 22/15°C. Floral buds of wild type self-compatible *B. napus* line “Westar” were emasculated 1 day before anthesis to avoid pollen contamination. The next day, stigma samples were collected by cutting the pistil just below the base of the stigma and immediately frozen in liquid nitrogen. By this method, stigma samples of “Westar” with no pollen pollinated (UP) and pollinated stigmas (PC2, PC5, PC10, PC20, PC30: self-pollinated stigmas of “Westar” at 2, 5, 10, 20, and 30 min; PI2, PI5, PI10, PI20, PI30: stigmas of “Westar” pollinated with the incompatible pollen of “W-3” at 2, 5, 10, 20, and 30 min) were collected.

### Transmission electron microscopy

The pollinated stigmas (PI30 and PC30) were vacuum-infiltrated and pre-fixed in a solution of 2.5% glutaraldehyde adjusted to pH 7.4 with 0.1 M phosphate buffer, fixed in 2% OsO_4_ in the same buffer, and then dehydrated and embedded in epoxy resin and SPI-812 (Structure Probe, Inc., http://www.2spi.com/), respectively. Ultra-thin sections were obtained using a Leica UC6 ultramicrotome (http://www.leica.com/) and were stained with uranyl acetate and subsequently with lead citrate. The observations and recording of images were performed using a Hitachi H-7650 transmission electron microscope (http://www.hitachi-hitec.com/) at 80 kV and a Gatan 832 CCD camera (http://www.gatan.com/).

### cDNA library construction and solexa/illumina sequencing

Total RNA was extracted using the DNA/RNA isolation kit (Qiagen). The quality and quantity of purified RNA were determined by measuring absorbance at 260 nm/280 nm (A260/A280) using a SmartSpec plus (BioRad). In total, 22 RNA samples (UP, PC2, PC5, PC10, PC20, PC30, PI2, PI5, PI10, PI20, and PI30, each with two biological replicates) were subjected to library construction using an Illumina® TruSeq™ RNA Sample Preparation Kit following the manufacturer's instructions. All samples were sequenced using an Illumina HiSeq 2500 sequencer at the National Key Laboratory of Crop Genetic Improvement, Huazhong Agricultural University.

### Sequence data analysis

Raw sequences were processed by removal of the 3' adaptor sequence, low-quality reads, and reads that are too short (less than 20 nt), leaving clean reads for subsequent analysis. All high-quality reads were mapped to the *B. napus* genome (Chalhoub et al., 2014) by TopHat v2.0.11 using the default parameters (Trapnell et al., 2009). Only uniquely mapped reads were considered for gene expression analysis. The program Cufflinks v2.2.0 was used to calculate differential gene expression and transcript abundance (Trapnell et al., 2010). Transcript abundance of each gene was estimated by FPKM. DEGs (differentially expressed genes) between UP and PC/PI samples were identified according to the restrictive conditions of an absolute value of log_2_ fold changes ≥ 1 and a FDR ≤ 0.01.

### Analysis and annotation of DEGs

Gene function annotation was performed in accordance with the method described by Wu et al. (2016). All *B. napus* genes (Chalhoub et al., 2014) were searched against the NCBI non-redundant (Nr) protein database using BlastP with an *E*-value ≤ 1E-05. GO terms associated with each BLAST hit were annotated using Blast2GO (Conesa et al., 2005). Then, all *B. napus* genes were searched against the InterPro database (http://www.ebi.ac.uk/interpro/) using InterProScan5 (Jones et al., 2014). Finally, *B. napus* genes were annotated by merging the annotation results of Blast2GO and InterPro. Blast2GO was also applied for GO enrichment analysis with a false discovery rate (FDR) ≤ 0.01, which can provide all GO terms that were significantly enriched in DEGs compared with the genome background.

### RT-PCR and qRT-PCR

Total RNA was extracted using the DNA/RNA isolation kit (Qiagen). Five micrograms of RNA was DNase-treated using a DNA-free kit (Ambion, http://www.ambion.com). First-strand cDNA synthesis was performed using a SuperScript kit (Gibco BRL, http://www.invitrogen.com). Real-time RT-PCR was also performed using a Bio-Rad IQ5 with SYBR Green detection (http://www.bio-rad.com/). *Actin* (Gene-Bank accession no.: AF111812) was used as an internal control to normalize transcript levels for all expression analyses. Supplemental Table S1 lists the specific primers used to test the genes.

### Accession numbers

Sequence data from this article can be found in the TAIR, NCBI (the NIH SRA) and *Brassica napus* Genome Resources (http://www.genoscope.cns.fr/brassicanapus/) data libraries.

## Results

### Comparative transcript profiling of compatible and incompatible reactions

**Figure 1:**
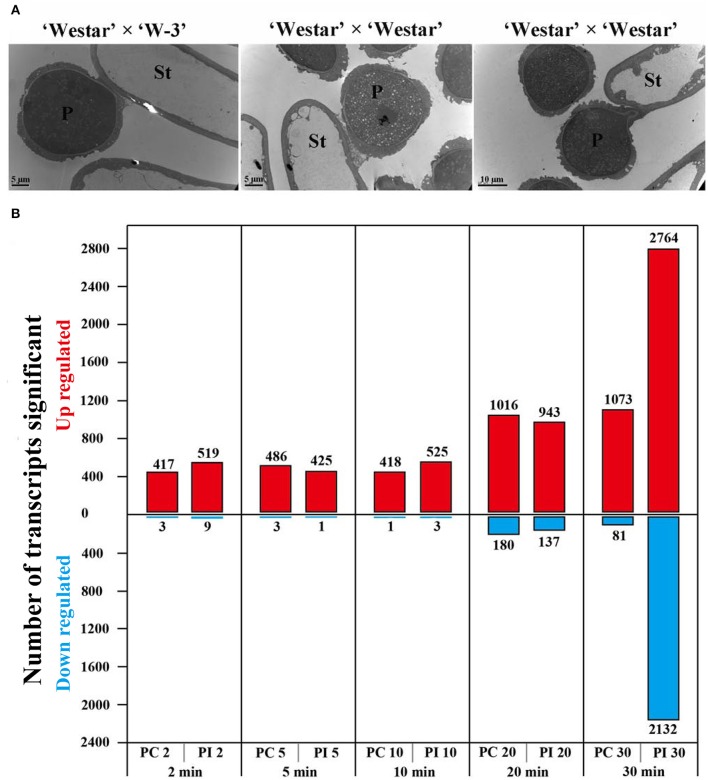
**Pollen-stigma interactions and DEGs (differentially expressed genes) identified in PC and PI samples. (A)** Transmission electron micrographs (TEM) of compatible and incompatible pollen-stigma interactions 30 min after pollination. In “Westar” × “W-3” (left, incompatible), pollen was (i.e., showed no change in morphology) intact in 6/6 samples. In “Westar” × “Westar” (middle and right, compatible), two patterns were observed in all the analyzed 5 samples (39 pollen grains): pollen intact (18 pollen grains); pollen germinated and beginning to invade the cell wall of the stigma papilla cell (eight pollen grains). P, pollen grain; St, stigma papilla cell; Bars = 5 μm in the left and middle pictures; 10 μm in the right picture. **(B)** Number of DEGs up- or down-regulated at different time points in UP vs. PC and UP vs. PI (log_2_ fold changes ≥ 1 and a FDR ≤ 0.01).

Transmission electron micrography (TEM) was used to compare SI and SC pollen-stigma interactions 30 min after pollination. When pollen of “W-3” was applied to the stigma of “Westar,” 23 pollen grains were observed being captured by the stigma papilla cell but there was no change in morphology of the pollen (Figure 1A, left panel). However, when “Westar” was self-pollinated, 39 pollen grains were captured and two kinds of pollen-stigma interaction patterns were observed. One pattern (Figure 1A, middle panel; 18 pollen grains) was similar to that observed in the “Westar” × “W-3” cross, with no change in morphology. The second pattern (Figure 1A, right panel; eight pollen grains) showed germination of the pollen tube and invasion of the cell wall of the stigma papilla cell. It could be deduced that it was possible for a compatible pollen grain to have experienced all initial steps of pollen-stigma interaction (adhesion, foot formation, hydration, germination and penetration) during the first 30 min following compatible pollination; incompatible pollen exhibited the first two steps in the same time period.

To explore the molecular mechanisms underlying compatible and incompatible pollen-stigma interactions, we employed Illumina (Solexa) sequencing technology to investigate the stigma transcriptome. Different kinds of stigma samples from wild type “Westar” were collected: un-pollinated stigmas (termed UP), stigmas pollinated with compatible pollen (PC) at multiple time points (2, 5, 10, 20, and 30 min, termed PC2, PC5, PC10, PC20, and PC30, respectively) and stigmas pollinated with incompatible pollen (PI) of “W-3” at the same time points as PC (termed PI2, PI5, PI10, PI20, and PI30, respectively). Compared with the genes expressed in UP, differential expression (log_2_ fold changes ≥ 1 and a FDR ≤ 0.01) analysis showed a moderate change of gene expression level in PC2, PC5, PC10, PI2, PI5, and PI10 (varying from 419 to 528 DEGs) and a drastic change in PC20, PC30, PI20, and PI30 (varying from 1080 to 4896 DEGs) (Figure 1B; Supplemental File S1). Based on the distribution of DEGs at each time point, we defined pollen-stigma interactions at 2, 5, 10 min as the “early stage pollination event,” and pollen-stigma interactions at 20 and 30 min as the “late stage pollination event,” relatively. At the early stage of pollination, 64 DEGs were up-regulated and only five were down-regulated (Figure 1B; Supplemental File S1). At the late stage of pollination, a drastic increase of DEGs was observed in UP vs. PI30 (2764 genes up-regulated and 2132 down regulated) (Figure 1B; Supplemental File S1), which further confirmed that pollen-stigma interaction already occurred by 30 min following pollination.

**Figure 2:**
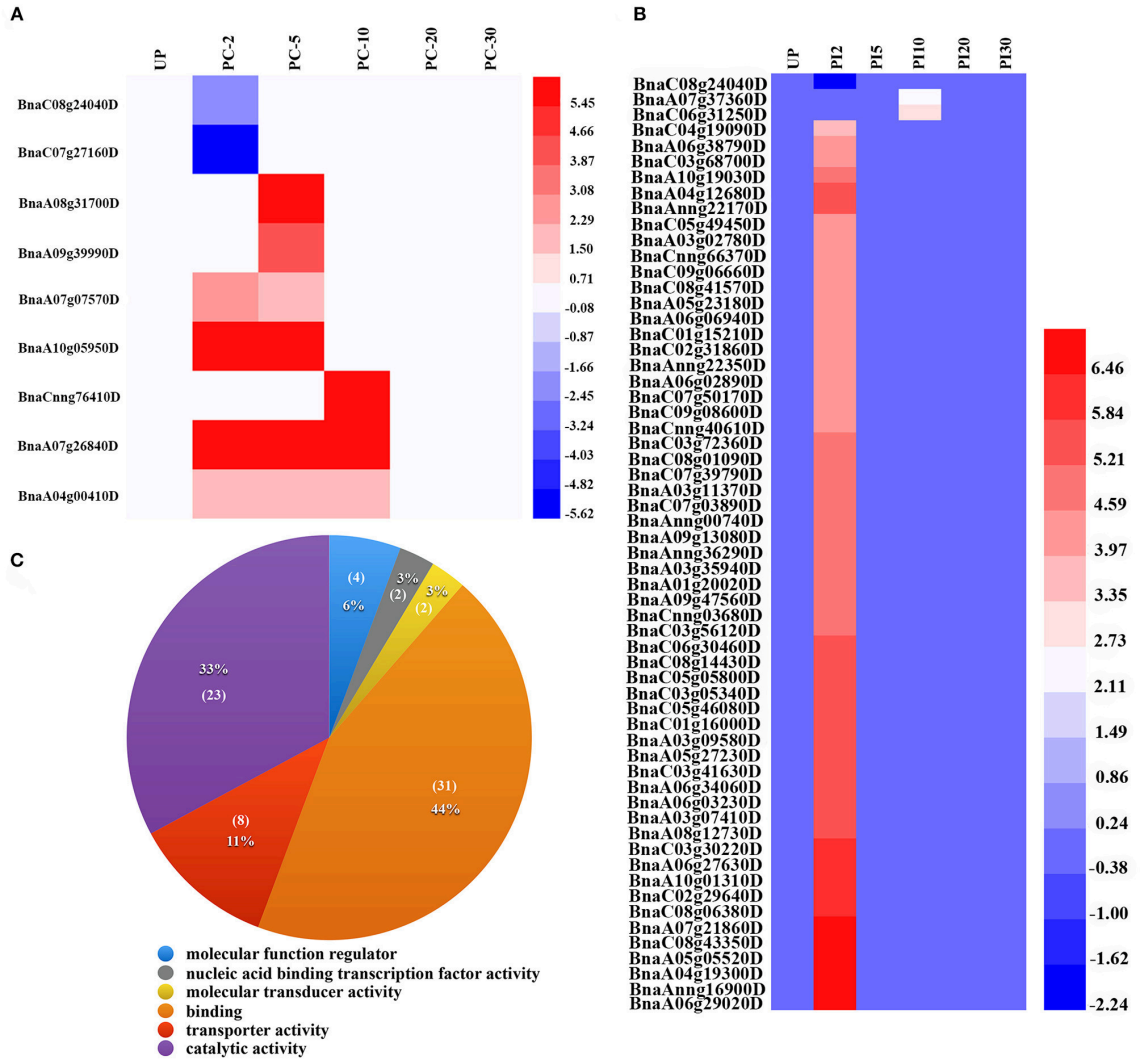
**Identification and annotation of early stage specific DEGs. (A)** Expression patterns of early stage specific DEGs in UP vs. PC. **(B)** Expression patterns of early stage specific DEGs in UP vs. PI. **(C)** GO annotation results of early stage specific DEGs in UP vs. PI.

A total of 1453 DEGs and 5071 DEGs were detected in all samples of UP vs. PC and UP vs. PI, respectively (Supplemental File S2). They were grouped by cluster analysis into nine distinct time-course clusters respectively according to their expression patterns with minor manual revision (Supplemental File S3). This classification revealed that only a small number of DEGs (nine in UP vs. PC and 60 in UP vs. PI) were early stage pollination specific (Figures 2A,B; Supplemental File S4). A moderate number of DEGs appeared during all stages of pollination and they were all up-regulated, with 529 DEGs in UP vs. PC and 542 DEGs in UP vs. PI (Supplemental File S5). The majority of DEGs were late stage specific, including 915 in UP vs. PC (704 up-regulated, 211 down-regulated) and 4469 in UP vs. PI (2337 up-regulated, 2132 down-regulated) (Supplemental File S6). The significant difference in the number of DEGs between UP vs. PC and UP vs. PI was mainly at the late stage of pollination with many more DEGs found in UP vs. PI, indicating that the signal transduction networks may be more complicated in UP vs. PI than UP vs. PC.

### Early stage specific DEGs

A total of 69 early stage specific DEGs were identified. Of the nine early stage-specific DEGs in UP vs. PC, seven were up-regulated and involved in pectinesterase activity (*BnaCnng76410D*), xylanase activity (*BnaA04g00410D*), chlorophyll binding (*BnaA07g07570D*) and other biological processes; the other two were down-regulated and involved in vacuolar sorting signal binding (*BnaC08g24040D*) and glucosidase activity (*BnaC07g27160D*) (Figures 2A,B; Supplemental File S7).

Among the 60 early stage specific DEGs in UP vs. PI, 59 genes were up-regulated with 57 found in UP vs. PI2 and two in UP vs. PI10 (Figure 2B). The most over-represented GO terms in molecular function were “binding” (GO:0005488) and “catalytic activity” (GO:0003824), accounting for 44% (31 genes) and 33% (23 genes) of the annotated terms, respectively (Figure 2C; Supplemental File S7). The term “binding” included genes related to phospholipid binding, metal ion binding, actin binding, DNA binding, ribonucleoside binding and tubulin binding. Of the 23 genes associated with catalytic activity, 11 were involved in protein serine/threonine kinase activity (GO:0004674). In addition, the terms “transporter activity” (GO:0005215), “molecular function regulator” (GO:0098772) and “transcription factor activity” (GO:0001071) were also identified (Figure 2C; Supplemental File S7). The only gene down-regulated was involved in vacuolar sorting signal binding (*BnaC08g24040D*). The majority of the DEGs at early stage in UP vs. PC and UP vs. PI were up-regulated, indicating that some biological processes might be activated by pollination; in the meanwhile, pollen expressed genes might contribute to the up-regulated DEGs.

### Late stage specific DEGs

**Figure 3:**
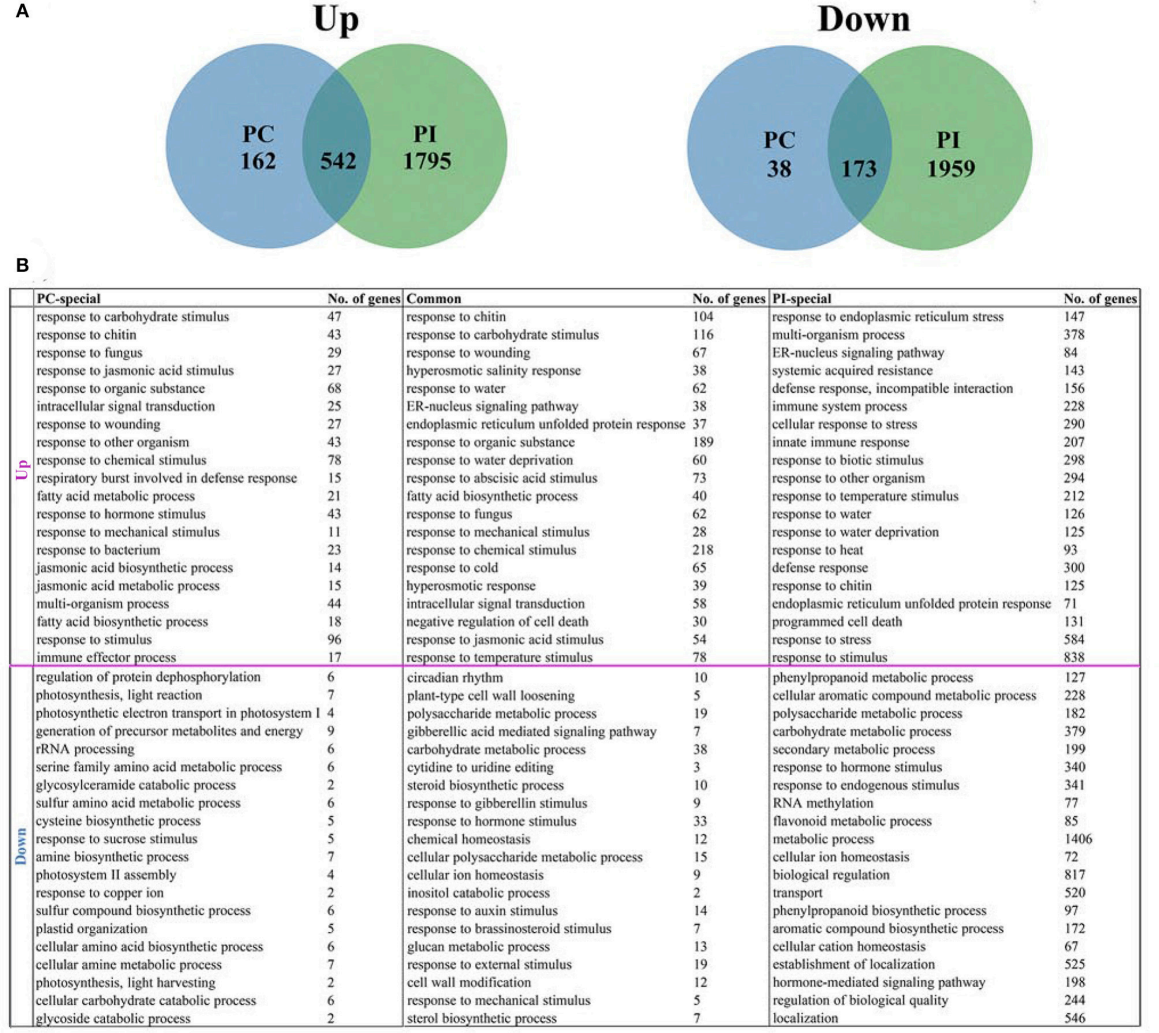
**Identification and annotation of late stage specific DEGs. (A)** Venn diagrams of late stage specific DEGs, comparing up- and down-regulated genes between UP vs. PC and UP vs. PI. **(B)** The 20 most highly represented GO terms for biological process in each DEG data set.

Among the up-regulated genes, 542 DEGs were shared by UP vs. PI and UP vs. PC, 1795 were UP vs. PI specific and only 162 were UP vs. PC specific. For down-regulated genes, 173 DEGs were shared in UP vs. PC and UP vs. PI, with 1959 specific in UP vs. PI and 38 in UP vs. PC (Figure 3A; Supplemental File S8). The biological functions of the DEGs were annotated and shown in Supplemental File S9. The 20 most highly represented GO terms of each DEG data set were listed in Figure 3B and Supplemental File S9.

For 162 genes up-regulated only in UP vs. PC, more than half of the over-represented GO terms were involved in stress response, such as responses to carbohydrate stimulus, chitin, fungus, and wounding. Besides, jasmonic acid related GO terms including response to jasmonic acid stimulus, jasmonic acid biosynthetic process and jasmonic acid metabolic process were found. Fatty acid metabolic process and fatty acid biosynthetic process were also identified in the annotation results. GO terms related to stress response were over-represented in genes up-regulated in both UP vs. PC and UP vs. PI. In addition, the terms “ER-nucleus signaling pathway,” “endoplasmic reticulum unfolded protein response,” “response to abscisic acid stimulus,” and “negative regulation of cell death” were also found. For genes up-regulated only in UP vs. PI, “response to endoplasmic reticulum stress” (147 genes), “multi-organism process” (378 genes) and “ER-nucleus signaling pathway” (84 genes) were the three most over-represented GO terms. In addition, defense response-related GO terms were also found, including “systemic acquired resistance,” “incompatible interaction,” “immune system process” and other processes. Stress response related GO terms were highly represented in all three DEG data sets for up-regulated genes, implying that pollen-stigma interactions might require elements involved in the processes of responding to stress.

For 38 genes down-regulated only in UP vs. PC, “regulation of protein dephosphorylation” was the most predominant GO term. GO terms involved in generation of precursor metabolites and energy, serine family amino acid metabolic process and cysteine biosynthesis were highly represented. GO terms related to photosynthesis, light reaction and photosynthetic electron transport in photosystem were also over-represented. GO terms of genes down-regulated both in UP vs. PC and UP vs. PI are mainly involved in polysaccharide metabolism (glucan metabolic process, plant-type cell wall loosening and cell wall modification) and response to hormone stimulus (by gibberellins, auxins and brassinosteroids). Besides, GO terms “circadian rhythm” and “carbohydrate metabolism” were also found in this category. GO terms of genes down-regulated only in UP vs. PI are mainly involved in metabolic processes, such as phenylpropanoid, cellular aromatic compound, polysaccharide, carbohydrate, secondary and flavonoid metabolism. A larger number of down-regulated genes were identified specifically in UP vs. PI and annotated to be related to metabolic processes, making the hypothesis possible that signaling transduction relying on some metabolic pathways might be cut off in incompatible response.

### DEGs at all stages of pollination

529 DEGs in UP vs. PC and 542 DEGs in UP vs. PI DEGs were found and up-regulated at all stages of pollination, including 457 genes in both UP vs. PC and UP vs. PI, 72 in UP vs. PC only, and 85 in UP vs. PI only (Supplemental File S10). The predominant biological process GO terms of DEGs up-regulated both in UP vs. PC and in UP vs. PI involved “plant-type cell wall modification,” “pollen tube growth,” and “pollination.” “Extracellular region,” “pollen tube,” and “plant-type cell wall” were the three most over-represented GO terms for cellular components. The most over-represented GO terms in molecular functions were “pectinesterase activity” and “hydrolase activity” (Supplemental File S11).

GO terms “pollen tube growth,” “reproductive process,” “extracellular region part,” and “plant-type cell wall modification” were also highly represented in UP vs. PC specific DEGs. (Supplemental File S11). The enriched GO term “pollination” (GO:0009856) was further analyzed, containing 21 of 72 UP vs. PC specific genes (Table 1) and several homologs of *Arabidopsis* genes involved in the pollination process. *ACA9* (*BnaA01g25310D*) was identified as a member of the ACA family; another member in this family, *ACA13*, was reported to function as an auto-inhibited Ca^2+^ transporter and was required for compatible pollination (Iwano et al., 2014). *ANXUR2* (*ANX2*), the homolog of *BnaA02g31020D*, was reported to function together with *ANXUR1* (*ANX1*) to regulate timing of rupture in pollen—pollen tubes of *anx1/anx2* mutants ruptured before arriving at the egg apparatus (Miyazaki et al., 2009). *LIP2* (homolog of *BnaA03g28130D*) and *LIP1* were anchored to the membrane in the pollen tube tip region via palmitoylation, which was essential for controlling pollen tube guidance into the micropyle (Liu et al., 2013). Ca^2+^-dependent protein kinase11 (*CPK11*) and *CPK24* (homolog of *BnaA04g19460D*) were involved in Ca^2+^-dependent regulation of the inward K^+^ (K^+^ in) channels in pollen tubes—disruption of *CPK11* or *CPK24* completely impaired Ca^2+^-dependent inhibition of K^+^ in currents and enhanced pollen tube growth (Zhao et al., 2013).

**Table 1:** **Enriched UP vs. PC specific DEGs in the GO term “pollination”**.

***Brassica napus*** **gene ID**	***Arabidopsis*** **gene ID**	**Gene name**	**Gene description**
*BnaA01g25310D*	*AT3G21180*	*ACA9*	Autoinhibited Ca^2+^-ATPase
*BnaA01g21610D*	*AT1G58122*	*CPUORF45*	Conserved upstream opening reading frame relative to major ORF AT1G58120.1
*BnaA02g07080D*	*AT5G59370*	*ACT4*	Belongs to the reproductive actin subclass that expressed in developing and reproductive tissues
*BnaA02g31020D*	*AT5G28680*	*ANX2*	Receptor-like kinase required for maintenance of pollen tube growth
*BnaA03g15420D*	*AT2G33420*	None	Unknown function
*BnaA03g28130D*	*AT3G02810*	*LIP2*	Receptor-like cytoplasmic kinase that controls micropylar pollen tube guidance
*BnaA03g37560D*	*AT3G24715*	None	Protein kinase superfamily protein with octicosapeptide/Phox/Bem1p domain
*BnaA04g18240D*	*AT3G24715*	None	Protein kinase superfamily protein with octicosapeptide/Phox/Bem1p domain
*BnaA04g19460D*	*AT2G31500*	*CPK24*	Member of Calcium Dependent Protein Kinase
*BnaA05g19830D*	*AT2G33420*	None	Unknown function
*BnaA07g08200D*	*AT3G21180*	*ACA9*	Autoinhibited Ca^2+^-ATPase
*BnaA07g19700D*	*AT1G29140*	None	Pollen Ole e 1 allergen and extensin family protein
*BnaA07g26300D*	*AT1G79860*	*ATROPGEF12*	Encodes a member of KPP-like gene family, homolog of KPP (kinase partner protein) gene in tomato
*BnaA08g18440D*	*AT1G67290*	*GLOX1*	Glyoxal oxidase-related protein
*BnaA09g30960D*	*AT1G28270*	*RALF4*	Member of a diversely expressed predicted peptide family showing sequence similarity to tobacco Rapid Alkalinization Factor (RALF)
*BnaC05g14980D*	*AT1G23540*	*PERK12*	Encodes a member of the PERK family of putative receptor kinases
*BnaC05g30810D*	*AT1G23540*	*PERK12*	Encodes a member of the PERK family of putative receptor kinases
*BnaC06g28370D*	*AT3G21180*	*ACA9*	Autoinhibited Ca^2+^-ATPase
*BnaC08g32350D*	*AT1G67290*	*GLOX1*	Glyoxal oxidase-related protein
*BnaC09g19870D*	*AT5G47000*	None	Peroxidase superfamily protein
*BnaCnng62360D*	*AT1G29140*	None	Pollen Ole e 1 allergen and extensin family protein

UP vs. PI specific DEGs were mainly involved in stress response and defense response (Supplemental File S11) and had totally different enriched GO-terms from UP vs. PC specific DEGs. As the mechanisms of SI and pathogen resistance (PR) are remarkably similar: SI involves recognition and rejection of “self” pollen grains and pathogen resistance (PR) involves recognition and rejection of pathogens (Hodgkin et al., 1988; Sanabria et al., 2008; Rea et al., 2010), thus the GO term “immune system process” (GO:0002376) was further analyzed and 21 of 85 UP vs. PI specific genes were identified (Table 2). *WRKY33* (a homolog of *BnaA03g17820D* and *BnaC03g21360D*) is a key transcriptional regulator of response to necrotrophic fungal pathogen *Botrytis cinerea* and *Alternaria brassicicola* infection in *Arabidopsis*, which involves cross-talk between jasmonate- and salicylate-regulated disease response pathways (Zheng et al., 2006; Birkenbihl et al., 2012). The expression of *AtCAF1A* (homolog of *BnaC03g54940D*) can be induced by multiple stress-related hormones and stimuli. Mutation of *AtCAF1A* caused defective deadenylation of stress-related mRNAs and reduced expression of pathogenesis-related (PR) genes *PR1* and *PR2*, making plants more susceptible to *Pseudomonas syringae pv*. tomato DC3000 (Pst DC3000) infection (Liang et al., 2009). *ERF4* (homolog of *BnaA03g33790D* and *BnaC03g39000D*) negatively regulates the expression of gene related to JA-responsive defense and the resistance to the necrotrophic fungal pathogen *Fusarium oxysporum* (McGrath et al., 2005). *JAZ1* (homolog of *BnaC08g36840D*), a member of the JAZ family, was a repressor of JA signaling pathways. *WRKY40* (homolog of *BnaA07g35260D* and *BnaC06g40170D*) was reported to regulate plant defense response in *Arabidopsis* in a complex pattern (Xu et al., 2006; Shen et al., 2007).

**Table 2:** **UP vs. PI specific DEGs involving the enriched GO term “immune system process”**.

***Brassica napus*** **gene ID**	***Arabidopsis*** **gene ID**	**Gene name**	**Gene description**
*BnaA01g05060D*	None	None	Unknown
*BnaA02g28700D*	*AT3G27210*	None	Unknown
*BnaA03g17820D*	*AT2G38470*	*WRKY33*	Member of the plant WRKY transcription factor family
*BnaA03g33790D*	*AT3G15210*	*ERF-4*	Encodes a member of the ERF (ethylene response factor) subfamily B-1 of ERF/AP2 transcription factor family (ATERF-4)
*BnaA03g55320D*	*AT5G06320*	*NHL3*	Encodes a protein whose sequence is similar to tobacco hairpin-induced gene (HIN1) and Arabidopsis non-race specific disease resistance gene (NDR1)
*BnaA05g19210D*	*AT3G19970*	None	Alpha/beta-Hydrolases superfamily protein
*BnaA06g12890D*	*AT1G18740*	None	Unknown
*BnaA06g21090D*	none	None	Unknown
*BnaA06g24410D*	*AT5G65530*	*RLCK VI_A3*	Encodes a protein kinase involved in mediating resistance to fungi and also trichome branch number
*BnaA07g15770D*	*AT3G52800*	Unknown	Unknown
*BnaA07g35260D*	*AT1G80840*	*WRKY40*	Pathogen-induced transcription factor
*BnaA08g30600D*	*AT4G32150*	*VAMP711*	Member of Synaptobrevin-like AtVAMP7C, v-SNARE (soluble N-ethyl-maleimide sensitive factor attachment protein receptors) protein family
*BnaC03g21360D*	*AT2G38470*	*WRKY33*	Member of the plant WRKY transcription factor family
*BnaC03g22390D*	*AT2G40000*	*HSPRO2*	Ortholog of sugar beet HS1 PRO-1 2 (HSPRO2)
*BnaC03g39000D*	*AT3G15210*	*ERF-4*	Encodes a member of the ERF (ethylene response factor) subfamily B-1 of ERF/AP2 transcription factor family (ATERF-4)
*BnaC03g54940D*	*AT3G44260*	*CAF1A*	Encodes one of the homologs of the yeast CCR4-associated factor 1
*BnaC05g09910D*	*AT1G13530*	None	Unknown
*BnaC05g50160D*	none	None	Unknown
*BnaC06g40170D*	*AT1G80840*	*WRKY40*	Pathogen-induced transcription factor
*BnaC08g24010D*	*AT2G36320*	none	Unknown
*BnaC08g36840D*	*AT1G19180*	*JAZ1*	Nuclear-localized protein involved in jasmonate signaling

### Genes enriched in all stigma samples

Three self-incompatibility related genes, the stigma determinant gene *BnSRK-1* (*BnaA07g25970D*) (Stein et al., 1991; Takasaki et al., 2000; Okamoto et al., 2007), pollen adhesion related genes *SLG* (*BnaA07g25960D*) and two copies of *SLR1* (*BnaC03g37350D* and *BnaA03g32070D*) (Luu et al., 1997, 1999) were found to be expressed highly in un-pollinated stigma, and the expression levels of these reported pollen-stigma interaction genes showed no obvious differences between compatible and incompatible pollinations. Thus, enriched genes in all stigma samples might also participate in pollen-stigma interactions. Genes with FPKM values above 250 in UP were selected, and 293 most highly expressed genes were identified and annotated (Supplemental File S12). Gene Ontology (GO) analysis and GO-term enrichment analysis were conducted to elucidate the biological functions of the stigma-enriched genes (Supplemental File S12). The GO term “recognition of pollen” (GO:0048544) was over-represented, with *BnSRK-1* (*BnaA07g25970D*), *SLG* (*BnaA07g25960D*) and *SLR1* (*BnaC03g37350D* and *BnaA03g32070D*) identified (Supplemental File S12).

**Figure 4:**
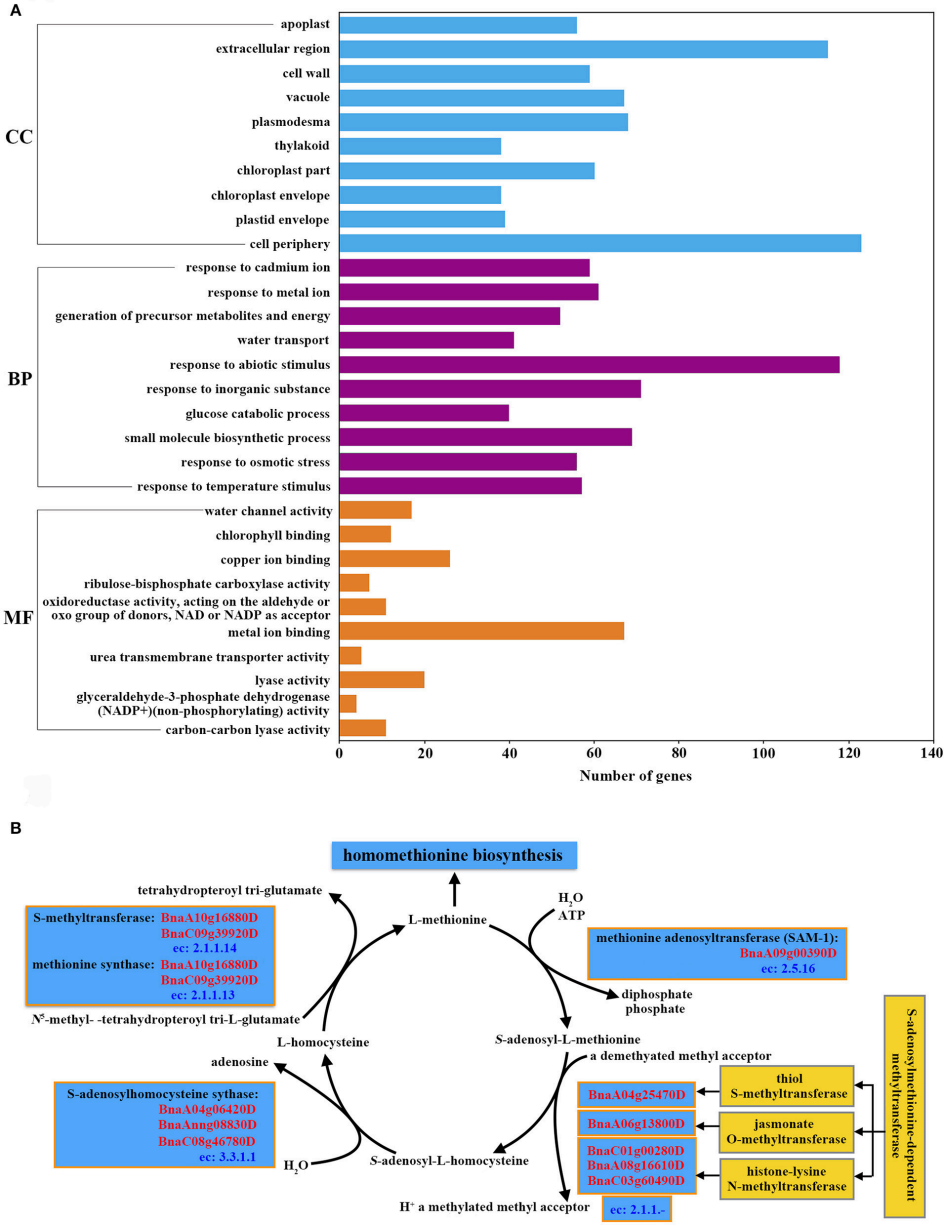
**Annotation of stigma-enriched genes. (A)** The 10 most highly represented GO terms in each category (biological process, cellular components and molecular functions). **(B)** Identification of the genes in the S-adenosyl-L-methionine (SAM) cycle and S-adenosylmethionine-dependent methyltransferases.

The 10 most highly represented GO terms in each category of biological process, cellular components and molecular functions are shown in Figure 4A. Several over-represented GO terms related to stress response were found, such as “response to abiotic stimulus” (osmotic stress and temperature stimulus), and “response to inorganic substance” (such as cadmium and metal ions) (Figure 4A; Supplemental File S12). GO term “cell wall” was identified in the category of cellular components. Plant cell wall is a highly dynamic, responsive structure that extends to the plasma membrane and underlying cytoskeleton during signal transduction (reviewed by Baluska et al., 2003). The cell wall of stigma is regarded as an obstacle for pollen tube growth and it also plays an important role in relaying information from external stimuli (reviewed by Humphrey et al., 2008). In addition, the terms “water transport” in molecular function and “water channel activity” in biological process were over-represented, confirming the role of stigma in providing resources for the hydration and germination of pollen grains in compatible pollination. Chloroplast related terms “thylakoid,” “chloroplast part,” and “chloroplast envelope” in cellular components, “chlorophyll binding” and “ribulose-bisphosphate carboxylase activity” in molecular functions were also found.

To characterize metabolic pathways activated in the stigma, stigma-enriched genes were mapped to metabolic pathways using the GO-EnzymeCode Mapping tool with the software Blast2Go. A total of 37 metabolic pathways were identified and ranked according to the number of mapped enzymes (Supplemental File S13). Starch and sucrose metabolism (eight enzymes including nine genes), biosynthesis of antibiotics (six enzymes, 16 genes) and cysteine and methionine metabolism (five enzymes, seven genes) were the three most over-represented metabolic pathways (Supplemental File S13). In addition to the results of metabolic pathway analysis, we also found that “adenosylhomocysteinase activity” (GO:0004013), “histone methyltransferase activity” (H3-K36 specific) (GO:0046975), “S-methyltransferase activity” (GO:0008172) and “methionine synthase activity” (GO:0008705) were over-represented in the GO-term enrichment analysis (Supplemental File S12). The identified five enzymes in the pathway of cysteine and methionine metabolism included all the four S-adenosyl-L-methionine (SAM) cycle related enzymes (Plant Metabolic Network, PMN, http://www.plantcyc.org/) (Figure 4B), implying that the active SAM cycle pathway may participate in the pollen-stigma interaction. Alongside the results of metabolic pathways analysis, the adenosylhomocysteinase activity (GO:0004013), histone methyltransferase activity (H3-K36 specific) (GO:0046975), S-methyltransferase activity (GO:0008172) and methionine synthase activity (GO:0008705) were also found to be over-represented (Supplemental File S12).

### Validation of RNA-seq data by quantitative real-time RT-PCR

**Figure 5:**
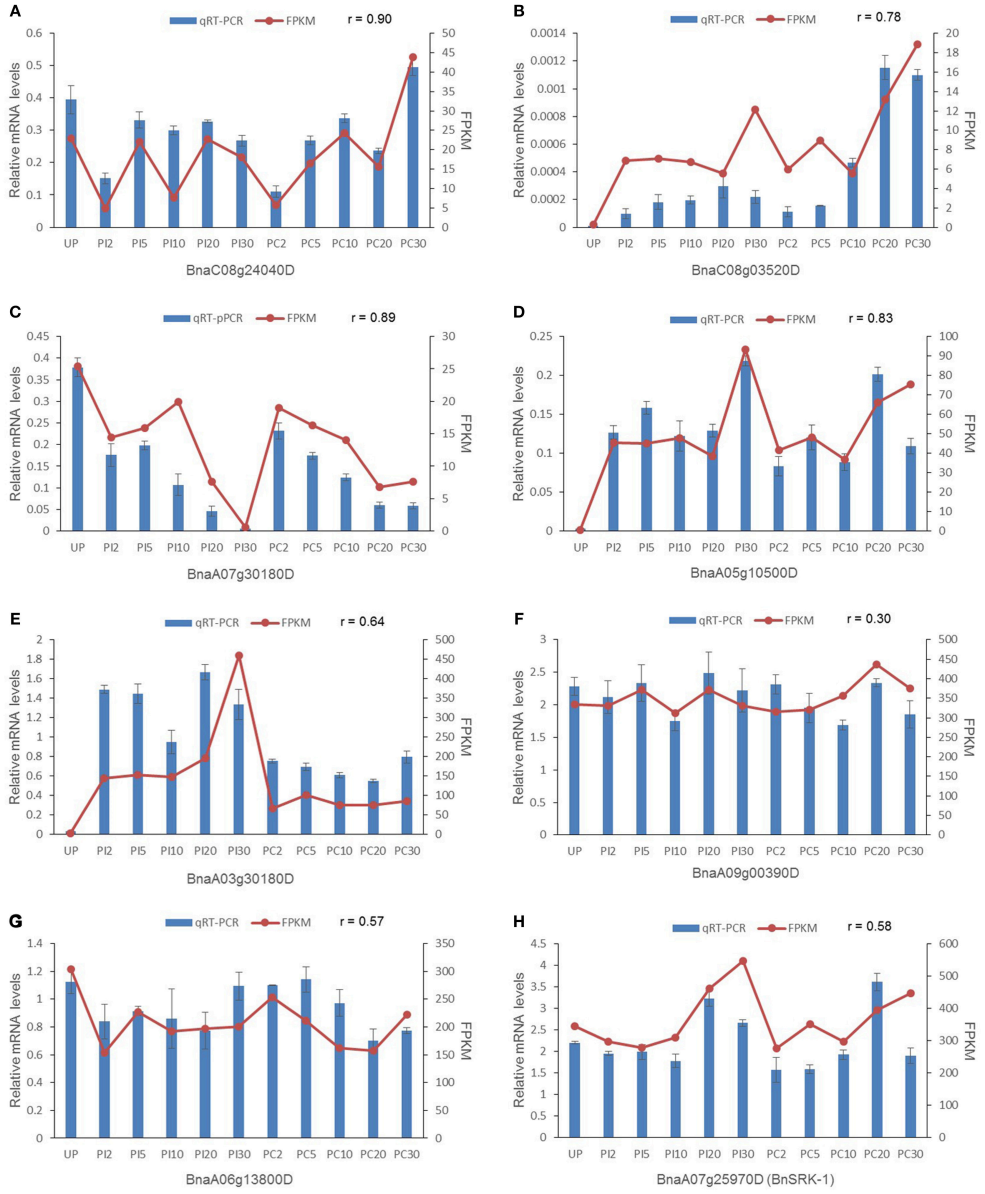
**Validation of eight randomly selected genes by qRT-PCR. (A)** One of the early stage DEGs. **(B,C)** Two DEGs at late stage. **(D,E)** Two DEGs at all stages. **(F–H)** Stigma-enriched genes. *BnaA09g00390D* and *BnaA06g13800D* are genes involved in SAM cycle. mRNA expression levels were normalized to the expression of *ACTIN*, and means from three biological replicates are shown. Error bars indicate ± SE. r represents the correlation coefficient.

To verify the DEGs and stigma-enriched genes identified by RNA-seq data, quantitative real-time RT-PCR was conducted with stigma samples harvested independently at the same time point as those collected for RNA-seq analysis. Five genes were selected randomly from DEGs at different stages, *BnSRK-1* and two genes enriched in all stigma samples involved in SAM cycle were chosen as well (Figure 5). The expression patterns of the DEGs analyzed by qRT-PCR were mostly consistent with the original RNA-seq data (a mean correlation coefficient of 0.81), few differences were found in the time points when gene expression level significantly changed (for example *Bna03g30180D*, Figure 5E), which was possibly caused by diverse sensitivities and algorithms between these two measuring means. The other three genes were expressed at high levels in all the samples and showed no significant difference in gene expression levels in each sample (Figures 5F–H). Their expression characteristics tested by qRT-PCR agreed well with those analyzed by RNA-seq, although low correlation coefficients were shown. These results indicated that the RNA-seq data were reliable.

## Discussion

### Transcriptional characteristics of pollen-stigma interactions

We have created one transgenic self-incompatible *B. napus* line “W-3” by complementing the function of *BnSP11-1* in self-compatible *B. napus* line “Westar” (Gao et al., 2016). There is a 3606-bp DNA fragment inserting into the promoter region of *BnSP11-1*, which is supposed to be responsible for the self-compatibility of “Westar” (Okamoto et al., 2007; Tochigi et al., 2011). “W-3” shows strong self-incompatibility and has the identical genetic background with “Westar” except for the induced functional *BnSP11-1*. Therefore, the transgenic *B. napus* line “W-3” was ideal to study compatible and incompatible pollen-stigma interactions. By observation of “Westar” stigmas 30 min after pollination using TEM, all “W-3” pollen grains were found to be intact (i.e., showed no change in morphology), while some “Westar” pollen grains germinated and began to invade the cell wall of the stigma papilla cell (Figure 1A). A time-course transcriptome analysis was employed to investigate compatible and incompatible pollen-stigma interactions, a moderate change in gene expression level was observed at 2, 5, and 10 min after pollination (varying from 419 to 528 DEGs), and a drastic change was found at 20 and 30 min after pollination (varying from 1080 to 4896 DEGs) (Figure 1B; Supplemental File S1). A moderate number of DEGs (529 in compatible interaction and 542 in incompatible interaction) appeared during all stages of pollination and they were all up-regulated; the majority of DEGs were detected at time points of 20 and 30 min, including 915 in compatible interaction (704 up-regulated, 211 down-regulated) and 4469 in incompatible interaction (2337 up-regulated, 2132 down-regulated). From the above results, it could be deduced that pollen-stigma interaction would complete 30 min after pollination, and downstream components were activated in signaling pathways of both compatible and incompatible responses, while the signal transduction networks in incompatible response might be more complicated than that in compatible response.

Enriched genes in all stigma samples including un-pollinated stigmas were firstly analyzed in our present study. We found the reported pollen-stigma interaction genes, the stigma determinant gene *BnSRK-1* (Stein et al., 1991; Takasaki et al., 2000; Okamoto et al., 2007), pollen adhesion related genes *SLG* and *SLR1* (Luu et al., 1997, 1999), were expressed highly in un-pollinated stigma and all pollinated stigmas, which is in accordance with the demonstration by Nasrallah (1974) that the SI response is regulated during stigma maturation: stigmas are initially compatible with self-pollen and acquire the ability to reject self-pollen in conjunction with anther dehiscence 1–2 days before flower opening or anthesis. Based on this feature, bud-pollination was developed by Shivanna et al. (1978), and has been used widely to amplify self-incompatible lines in *Brassica*. A total of 37 metabolic pathways were identified. Starch and sucrose metabolism is one of the three most over-represented metabolic pathways, which has been reported by Matsuda et al. (2014). Biosynthesis of antibiotics, and cysteine and methionine metabolism pathways are first annotated in this study. Genes involved in cysteine and methionine metabolismwere tested by qRT-PCR and shown to be expressed at high levels in each sample. Further researches on other enriched genes might reveal genes necessary for pollen-stigma reaction signaling pathways.

Genes expressed differentially in compatible or incompatible reactions at all-time points compared to un-pollinated stigmas were also firstly identified in this study. A moderate change of gene expression was observed at 2, 5, and 10 min after pollination, but only nine in compatible and 60 in incompatible reactions were early stage specific DEGs. Quite limited genes expressed in pollen might be included because there was only one gene shared by UP vs. PC and UP vs. PI at early stage, and genes abundant in pollen would not lead to such a limited number of early stage DEGs in compatible reaction. For the 60 DEGs incompatible reaction, the most over-represented GO terms in molecular function were “binding” and “catalytic activity,” accounting for 44% (31 genes) and 33% (23 genes) of the annotated terms, respectively. The term “binding” included genes related to phospholipid binding, metal ion binding, actin binding, DNA binding, ribonucleoside binding and tubulin binding. A subunit of the microtubule (MT) network, alpha 2–4 tubulin, was identified in the proteomic analysis for self-incompatibility response in *Brassica napus* (Samuel et al., 2011). It was proposed that the depolymerization of MT is necessary for accepting compatible pollen. Thus, genes related to tubulin binding might regulate self-pollen rejection by affecting the stability of MT network. In addition, compared to previous studies, a quantity of DEGs were also represented here, such as genes related to jasmonic acid production and plant-type cell wall metabolism were up-regulated at late stage in UP vs. PC samples, which is consistent with results reported by Swanson et al. (2005) and Matsuda et al. (2014). Most genes involved in stress response or defense response were up-regulated in both incompatible and compatible reactions at late-time points (20 and 30 min after pollination), while the unique proteins in the same GO terms were found to be down-regulated in incompatible pollination previously (Samuel et al., 2011). There is no discrepancy between this study and the previous one, since the previous study focused more on degraded proteins that were possibly ubiquitinated (Samuel et al., 2011).

### Pollen-stigma interactions and pathogen-plant interactions

Compatible pollen can penetrate the cell wall of papillae cells while incompatible pollen-stigma interactions can cause blocks in pollen hydration, germination and pollen tube growth at the stigma. However, the downstream molecular events were largely unknown. In pathogen-plant interactions, a classic “zigzag” model was proposed to illustrate this process, which involves two branches of the plant immune system: PTI (PAMP-triggered immunity, inducing responses activated upon recognition of conserved PAMPs, such as peptidoglycan, flagellin, chitin and others) and ETI [effector-triggered immunity, inducing R (resistance) gene-mediated resistance reactions activated upon recognition of an avirulence factor] (Chisholm et al., 2006; Jones and Dangl, 2006). First, PAMPs (pathogen-associated molecular patterns) can be perceived by plants, inducing PTI which can stop the colonization of pathogens. Then the pathogens can adapt the effectors that contribute to pathogen virulence to interfere with PTI and induce effector-triggered susceptibility (ETS), permitting successful invasion of the plant cells. However, if the plant contains an R protein which can specifically recognize the effector, then ETI is induced, preventing the pathogen from invading the plant cells. In partial summary, two contrary interaction patterns (compatible and incompatible) occur both in pollen-stigma interactions and pathogen-plant interactions.

Close parallels between SI and plant–pathogen interactions have been suggested (Hogenboom, 1983; Hodgkin et al., 1988; Nasrallah, 2005; Sanabria et al., 2008), both involving recognition and rejection, albeit of genetically similar (“self”) pollen grains vs. “non-self” pathogens. It is hypothesized that both SI and plant–pathogen interaction processes may share the same basal genetic defense network, and genes involved in SI and defense might have common ancestors (Rea et al., 2010; reviewed by Sanabria et al., 2008). In addition, both SI and disease resistance signaling pathways were triggered by interactions between small peptide ligands (located in pollen or pathogen) and plasma membrane-spanning receptor kinases. We speculate that close parallels between SC and plant–pathogen interactions (mainly effector-triggered susceptibility, ETS) may also exist. Both processes comprise the recognition of extracellular materials (pollen/pathogen) and penetration into the “host” by a tubular cell emanating from a spore-like structure. Defense-related genes might function not only in defense against pathogens, but also in response to pollination (Tung et al., 2005). In rice, many stigma-specific genes encode stress and defense related proteins and stigma-specific genes shared some common cis-regulatory elements (GCC box for example) with stress-responsive genes (Li et al., 2007). In our annotation results of late stage specific DEGs, of the 20 most over-represented GO terms, stress response related ones appeared in all three DEG data sets: genes up-regulated only in UP vs. PC, genes up-regulated only in UP vs. PI and genes up-regulated both in UP vs. PC and UP vs. PI (Figure 3B; Supplemental File S9). Especially in the genes up-regulated only in UP vs. PC, more than half of the most over-represented GO terms were involved in stress response, such as responses to carbohydrate stimulus, chitin, fungus, wounding and others (Figure 3B; Supplemental File S9). However, in the genes up-regulated only in UP vs. PI, defense response related GO terms were over-represented, such as systemic acquired resistance, incompatible interaction, immune system process and others (Figure 3B; Supplemental File S9), which supports the hypothesis that SI and pathogen-plant interactions showed some common signaling pathways. Also in the DEGs found in all stages of pollination, stress and defense response related GO terms were over-represented in UP vs. PI specific genes but not in UP vs. PC specific genes (Supplemental File S11). We speculated that in pollen-stigma interactions, the stigma can recognize components located on the compatible pollen coat (just like PAMPs in the pathogen) and induce the stress response, a process similar to PTI and ETS. But when incompatible pollen is applied, the stigma can recognize components located on the pollen coat and the SP11/SCR protein (just like effectors in the pathogen), inducing both the stress and defense responses, a process similar to PTI and ETI.

### S-adenosyl-L-methionine (SAM) cycle in stigma

Genes most highly expressed in stigmas consistently showed no obvious differences in the expression levels between compatible and incompatible pollinations, but they may also play important roles in pollen-stigma interactions, possibly through a means of post-translational modification such as phosphorylation, glycosylation, or methylation. In our expression data, several genes implicated in pollen-stigma interactions were found, including self-incompatibility gene *BnSRK-1* (*BnaA07g25970D*) (Stein et al., 1991; Takasaki et al., 2000; Okamoto et al., 2007), pollen adhesion related gene *SLG* (*BnaA07g25960D*) and two copies of *SLR1* (*BnaC03g37350D* and *BnaA03g32070D*) (Luu et al., 1997, 1999) (Supplemental File S12). We inferred that more unknown genes abundant in stigma may be required for pollen-stigma interactions. By analyzing the involved metabolic pathways of the stigma enriched genes, we found that the pathway of cysteine and methionine metabolism was over-represented, and all the four S-adenosyl-L-methionine (SAM) cycle related enzymes were found in this pathway (Figure 4B; Supplemental File S13). In the SAM cycle, S-methyltransferase and methionine synthase (*BnaA10g16880D* and *BnaC09g39920D*) were responsible for the generation of methionine. Then methionine adenosyltransferase (*SAM-1, BnaA09g00390D*) can convert methionine to SAM which is a methyl donor used for many cellular transmethylation reactions. In the transmethylation reactions SAM is converted to S-adenosyl-homocysteine (SAH) under the catalysis of S-adenosylmethionine-dependent methyltransferases, and three of them were identified in our data (*BnaA04g25470D, BnaA06g13800D, BnaC01g00280D, BnaA08g16610D*, and *BnaC03g60490D*). Finally S-adenosylhomocysteine synthase (*BnaA04g06420D, BnaAnng08830D*, and *BnaC08g46780D*) can convert SAH to homocysteine which was the precursor of methionine. SAM is then regenerated from methionine to finish the cycle (Figure 4B) (Giovanelli et al., 1985). Methionine synthase 1 (*MS1, BnaA10g16880D*, and *BnaC09g39920D*) plays a key role for the continual reactions of SAM cycle, which catalyzes the last reaction in *de novo* Met synthesis and helps to regenerate the methyl group of AdoMet following methylation reactions (Ravanel et al., 2004). Interestingly, *BnMS1* (also named *ATCIMS*, ortholog of *At5g17920*) was identified among the 19 down-regulated proteins following the SI reaction in *B. napus* as well (Samuel et al., 2011), indicating that *BnMS1* might play a role in regulating compatible and incompatible reactions.

## Author contributions

TZ and CG designed and performed the research, analyzed data, and wrote the article with contributions of all the authors; YAY and ZL performed research and analyzed data; GZ, YOY, ZD, and BL provided technical assistance to TZ and CG; CM, JW, BY, JS, JT, and TF supervised the experiments; CM supervised and complemented the writing.

## Funding

This work was funded by grants from the National Key Research and Development Program of China (No. 2016YFD0101301), China Postdoctoral Science Foundation (2014M552055; 2015T80816), National Natural Science Foundation of China (3157101052).

### Conflict of interest statement

The authors declare that the research was conducted in the absence of any commercial or financial relationships that could be construed as a potential conflict of interest.
