# Supplementary material for: Time-Course Transcriptome Analysis of Compatible and Incompatible Pollen-Stigma Interactions in Brassica napus L
Source: Front Plant Sci. 2017 May 3;8:682. doi: 10.3389/fpls.2017.00682 (PMC5413569; doi:10.3389/fpls.2017.00682)
Supplement: Supplementary file 1 [file Table1.DOCX]

**Supplemental Table S1. Primers for quantitative RT-PCR verification of RNA-seq data**

| Gene | Forward primer | Reverse primer |
| --- | --- | --- |
| BnaC08g24040D | GGGTTTGGTGGGACTATGTG | TTTGCCAACCTGAGACTCCT |
| BnaC08g03520D | CCTCCTCCCACAAAATCTGA | CGCTTGACACGAATACGAAG |
| BnaA07g30180D | TCAACGGATCCTCCTTCTTG | CTTGCCAACATCAGCTTCAA |
| BnaA05g10500D | CGCCTTCTCTTGGATTCTTG | GCTGTGACTAGAGGCGGTTC |
| BnaA03g30180D | CTTGCCAACATCAGCTTCAA | CTCACGTTTGAGCACTGGAA |
| BnaA09g00390D | AAGACCAGACGGGAAGA | TTGCCTCACGATGTAGG |
| BnaA06g13800D | CGCAGGAGCCTTCACTT | GAAACGCCGAGAAATCG |
| BnaA07g25970D | GCATGGAGAAATTGGAAGGA | CACAGACGACATGGTTGGTC |
