# Supplementary material for: Time-Course Transcriptome Analysis of Compatible and Incompatible Pollen-Stigma Interactions in Brassica napus L
Source: Front Plant Sci. 2017 May 3;8:682. doi: 10.3389/fpls.2017.00682 (PMC5413569; doi:10.3389/fpls.2017.00682)
Supplement: Supplementary file 2 [file DataSheet1.DOCX]

Supplementary Material

Time-course Transcriptome Analysis of Compatible and Incompatible Pollen-Stigma Interactions in *Brassica napus L.*

Changbin Gao^#^, Tong Zhang^#^, Zhiquan Liu, Yao Yue, Chaozhi Ma*, Jun Chen, Wen Zhai, Guilong Zhou, Yong Yang, Kaining Hu, Zhiqiang Duan, Ya Li, Bing Li, Jijun Li, Jing Wen, Bin Yi, Jinxiong Shen, Jinxing Tu and Tingdong Fu

^#^These authors contributed equally to this work.

*** Correspondence:** Chaozhi Ma: yuanbeauty@mail.hzau.edu.cn

# Supplementary Data

**Supplemental File S1.** DEGs between UP and PC, PI at different time points.

**Supplemental File S2.** All the DEGs in PI and PC.

**Supplemental File S3.** Clusters of the DEGs in PC and PI.

**Supplemental File S4.** Early stage pollination specific DEGs in PC and PI.

**Supplemental File S5.** DEGs in the all stages in PC and PI.

**Supplemental File S6.** Late stage pollination specific DEGs in PC and PI.

**Supplemental File S7.** Annotation of the early stage specific DEGs.

**Supplemental File S8.** Comparation of the late stage specific DEGs.

**Supplemental File S9.** Annotation of the late stage specific DEGs.

**Supplemental File S10.** Comparation of the DEGs in the all stages of pollination.

**Supplemental File S11.** Annotation of the DEGs in the all stages of pollination.

**Supplemental File S12.** Annotation of the stigma-enriched genes.

**Supplemental File S13.** Metabolic pathways of the stigma-enriched genes.

**Supplemental Table S1.** Primers for quantitative RT-PCR verification of RNA-seq data.
